# ICTUSCOG: Poststroke cognitive impairment following nondisabling stroke: Study protocol of a spanish multicentre prospective cohort study

**DOI:** 10.1371/journal.pone.0330255

**Published:** 2025-09-09

**Authors:** Cristina Moreno-Loscertales, Paula Canasto-Jiménez, Mario Bautista-Lacambra, Marta Serrano-Ponz, Isabel Campello-Morer, Marta Palacín-Larroy, Herbert Tejada-Meza, Esther Sierra-Martínez, Jorge Navarro López, Fidel Alfaro-Almagro, Gonzalo Forno-Martinic, Miguel Ángel Marín-Cárdenas, Patricia de la Riva, Raquel Laspiur Gándara, Eugenia Marta-Moreno, Javier Marta-Moreno

**Affiliations:** 1 Department of Neurology, Hospital Universitario Miguel Servet, Zaragoza, Spain; 2 Neuroscience Research Group, Instituto de Investigación Sanitaria Aragón, Hospital Universitario Miguel Servet, Zaragoza, Spain; 3 Neuropsychology Department, San Juan de Dios Hospital, Zaragoza, Spain; 4 Grupo Decisión Multicriterio Zaragoza. Department of Applied Economics, University of Zaragoza, Zaragoza, Spain; 5 Wellcome Centre for Integrative Neuroimaging, FMRIB, Nuffield Department of Clinical Neurosciences, University of Oxford, Oxford, United Kingdom; 6 Geroscience Center for Brain Health and Metabolism (GERO), Santiago, Chile; 7 Neuropsychology and Clinical Neuroscience Laboratory (LANNEC), Physiopathology Department – ICBM, Neuroscience and East Neuroscience Departments, Faculty of Medicine, University of Chile, Santiago, Chile; 8 Department of Radiology, Miguel Servet University Hospital, Zaragoza, Spain; 9 Neurology Department, Donostia University Hospital, San Sebastian, Spain; 10 Stroke Research group, Biogipuzkoa Health Research Institute, San Sebastian, Spain; University of Thessaly Faculty of Medicine: Panepistemio Thessalias Tmema Iatrikes, GREECE

## Abstract

**Background:**

Stroke is a leading cause of death and disability globally, with frequent cognitive sequelae affecting up to 60% of stroke survivors. Despite the high prevalence of post-stroke cognitive impairment (PSCI), early detection remains underemphasized in clinical practice, with limited focus on broader neuropsychological and affective symptoms. Stroke elevates dementia risk and may act as a trigger for progressive neurodegenerative diseases. However, the underlying neurobiological mechanisms and the interaction between vascular and degenerative pathways are poorly understood. The ICTUSCOG study aims to address these gaps by determining the incidence, predictors and progression factors of PSCI in a prospective, multicenter cohort of nondisabling stroke patients. The work will explore distinct patient profiles, evaluate the role of biomarkers, and develop a predictive model to identify at risk individuals.

**Methods:**

ICTUSCOG is a five-year observational project involving four Spanish centres. Recruitment began in 2022 and includes consecutive patients aged 18–75 with no prior cognitive impairment and nondisabling stroke. Participants undergo detailed neuropsychological, functional, and neuroimaging assessments at baseline, 3, 6, and 12 months, and annually thereafter. Key data include stroke characteristics, vascular risk factors, advanced neuroimaging metrics, and biological biomarkers. Neuropsychological assessments incorporate domain-specific validated tools tailored for stroke patients.

**Discussion:**

The study will quantify the incidence of early and late PSCI, identify predictors of progression, and characterise cognitive profiles. Multivariate models and clustering techniques will explore interactions among clinical, biological and imaging data. A predictive model will be developed and validated for clinical use. ICTUSCOG will provide critical insights into the mechanisms and trajectories of PSCI, informing prevention, early intervention, and rehabilitation strategies. The work aims to establish predictive tools and care pathways to mitigate the burden of cognitive impairment in stroke survivors.

## Introduction

### Background

Poststroke cognitive impairment (PSCI) refers to the decline in mental function following an overt stroke (ischemic or hemorrhagic) and encompasses a spectrum ranging from mild cognitive impairment (MCI) to dementia (PSD) [1]. More generally, vascular cognitive impairment (VCI) refers to all forms of cognitive dysfunction linked to cerebrovascular disease [2].

Depending on the diagnostic and study methodologies employed, prevalence differs widely: in patients without pre-existing cognitive impairment up to 20% will develop PSD but milder deficits are much more common [2,3]; 83% of stroke survivors show impairment in at least 1 cognitive domain whereas 50% of patients are impaired in multiple (≥3) domains when tested 3 months post-event, which serves as a predictor of dementia, dependency, and mortality [4].

The prevalence of PSD is increasing; this is probably due to the rising life expectancy of the population, advancements in reperfusion therapies, and improved post-stroke care. These factors have contributed to a decline in stroke mortality and an increase in the number of individuals recovering with sequelae that are, at first glance, nondisabling [5]. Despite this data, standard clinical scales lack elements that assess cognitive domains. Historically, emphasis has been placed on motor and physical aspects and there has been little focus on the affective and cognitive spheres. Screening for cognitive deficits during follow-up receives disproportionately less attention than other neurological sequelae. As a result, an important factor that critically influences functional prognosis is often overlooked.

It seems clear that stroke increases the risk of dementia, but not all individuals who experience a stroke will develop it. Certain factors related to disruption of the blood-brain barrier [6], cerebral hypoperfusion and cerebrovascular pathology itself heighten this risk. However, the role of other concurrent predictors is uncertain. Comorbidities, particularly cardiovascular and renal disease, diabetes, metabolic syndrome, cancer, dysimmune or chronic inflammatory diseases, and the role of the Apolipoprotein E (ApoE) genotype are not well-defined.

From a clinical perspective, we are aware that care pathways are inadequate for detecting PSCI. Early testing is known to predict long-term outcomes, functional recovery and early mortality, and should be part of routine assessment [7,8]. One of the objectives of this study is to document care practices in our setting and evaluate functional and social outcomes, aligning with global efforts such as the World Health Organization’s Brain Health initiative and the American Academy of Neurology’s Brain Health Program [9,10].

PSCI is a complex condition involving overlapping vascular and neurodegenerative processes which seem to interact and coexist resulting in cumulative brain damage [5]. The current perspective suggests that risk factors and comorbidities triggering vascular pathology are also present in the cascade of Alzheimer’s pathology and other neurodegenerative disorders, with most cases being mixed in nature [7]. Copathology with subclinical Alzheimer’s disease (AD) may be a major determinant of PSD. The consolidation and availability of biomarkers for AD have advanced research in this area but findings remain inconclusive.

We hypothesize that at least two distinct mechanisms underlie PSCI: one primarily vascular, with stable or limited progression, and another involving a progressive decline due to concurrent neurodegenerative disease. The temporal and bidirectional nature of this interaction is still not well defined. A long-term cohort is essential to distinguish between these trajectories.

### Study aims

The ICTUSCOG study aims to prospectively identify clinical, lesional, biological, and pharmacological factors associated with PSCI through the long-term follow-up of a cohort of stroke patients. To this end, the following objectives were set:

- To determine the early incidence of PSCI by identifying patients diagnosed during evaluations conducted at 3 and 6 months post-stroke.
- To assess the late incidence of PSCI by detecting patients who present PSCI during assessments conducted 12 months or more after the stroke.
- To identify predictors of cognitive decline and factors associated with progression, with a specific focus on biological and neuroimaging markers to investigate the interplay between vascular pathology, comorbidities, and underlying neurodegenerative processes.
- To explore the existence of different patient profiles that are more likely to experience PSCI.
- To examine the potential predictive role of pro-inflammatory states, metabolic syndrome, and ApoE4 genotype.
- To characterize neuropsychological profiles of affected individuals, assess their relationship with stroke characteristics and determine whether specific patterns predict progression or coexistence with AD.
- To estimate the prevalence of post-stroke depression and apathy and identify associated risk factors.
- To evaluate whether predictive factors differ between early and late cognitive impairment or dementia (within the first 3 and 6 months versus beyond these time frames), particularly regarding mixed pathology involving both AD and vascular contributions.

These aims will enhance understanding of the complex mechanisms underlying post-stroke cognitive outcomes and will guide the development of a predictive model for clinical use, enabling early identification and targeted intervention to reduce the burden of PSCI.

This initiative is aligned with the STROKOG Consortium (founded in 2017 under The International Society of Vascular Behavioural and Cognitive Disorders), which promotes the use of harmonized diagnostic criteria and standardized assessments. Participation in this international collaborative network allows us to address broader research questions and facilitates collaborative projects.

## Materials and methods

### Design and setting of the study

ICTUSCOG is a prospective, multicenter, observational cohort study with a five-year follow-up. Patients will be consecutively recruited from specialized Stroke Units across four centres: Miguel Servet University Hospital, Doce de Octubre University Hospital, Donostia University Hospital and Navarra University Hospital. Recruitment began in February 2022 and will continue until recruitment targets are met. Participants are enrolled during the acute phase (within 72 hours post-stroke) and undergo comprehensive assessments at baseline, 3, 6, 12 months and annually thereafter over a five-year period with data collection at predefined intervals. All consecutively admitted patients will be screened for eligibility, and those meeting the inclusion criteria will be invited to participate in the study. Informed consent will be obtained prior to the collection of data and biological samples. No experimental intervention is applied; all participants will receive standard care in accordance with local stroke management guidelines.

### Sample size calculation

The sample size calculation was based on an estimated 40% prevalence of PSCI at one year, according to existing literature and preliminary data from the initial cohort inclusions. With a 95% confidence level, a sample size of 97 participants was deemed necessary to achieve an approximate 10% margin of error. To account for an expected attrition rate of 25% during follow-up, at least 130 participants will be recruited to ensure sufficient power for the primary analysis (comparison of proportions or means between patients with and without PSCI). Exploratory multivariate analyses (logistic regression and predictive modeling) are planned as secondary and descriptive, acknowledging that their statistical power will be limited by the number of observed events and the overall sample size.

### Patient enrolment

#### Participants.

Subjects aged 18–75 years who have experienced a first-ever mild-to-moderate stroke (National Institutes of Health Stroke Scale (NIHSS) ≤ 8), defined as an acute focal neurological deficit, combined with: (1) an acute ischemic infarction documented by a positive Difusión-weighted imaging (DWI) lesion on Magnetic Resonance Imaging (MRI) or (2) an intracerebral haemorrhage documented by computed tomography (CT) scan or MRI within the last 72 hours will be invited to participate. Eligible individuals must have no prior cognitive impairment, confirmed through a thorough review of the patient’s medical history and an interview with both the patient and a close informant (with at least weekly contact and detailed knowledge of the patient’s cognitive and functional status). Additionally, the shortened Spanish version of the Informant Questionnaire on Cognitive Decline in the Elderly (SS-IQCODE) [11] will be administered to the informant to assess the patient’s condition immediately prior to the stroke compared to one year earlier and must have a pre-admission modified Rankin Scale (mRS) score of 0–1 [12]. Patients must be fluent in Spanish, able to provide informed consent and willing to participate in follow-up assessments.

Exclusion criteria include any prior stroke, even if fully recovered and without sequelae (silent strokes identified through neuroimaging do not constitute exclusion criteria), severe aphasia that could interfere with neuropsychological assessment, diagnosis and treatment for depression prior to stroke or any severe psychiatric disorder, dependence on or treatment with psychoactive substances, diagnosis of epilepsy under active treatment (within the past 5 year), any of the following vascular conditions: cerebral venous thrombosis, traumatic cerebral haemorrhage, intracerebral haemorrhage due to vascular malformation, subarachnoid haemorrhage, intraventricular haemorrhage or any other neurological disease that could lead to cognitive impairment or brain damage. Severe comorbid conditions limiting life expectancy (< 2 years) or scheduled for major surgery under general anaesthesia within the next 6 months will also constitute exclusion criteria.

A patient will be considered eligible for inclusion in the ICTUSCOG cohort if they have a follow-up at 3 months and at least one additional follow-up at 6 and 12 months. Included patients will then receive follow-up visits annually, up to 5 years.

### Data collection and assessments

Study data will be collected and managed using Research Electronic Data Capture (REDCap) tools hosted at the Institute for Health Sciences of Aragon [13,14]. REDCap is a secure, web-based software platform designed to support data capture for research studies, providing: 1) an intuitive interface for validated data capture; 2) audit trails for tracking data manipulation and export procedures; 3) automated export procedures for seamless data downloads to common statistical packages; and, 4) procedures for data integration and interoperability with external sources.

The assessments will be collected at the various in-person visits conducted during the hospital stay and at follow-up visits at 3, 6, 12 months and annually, up to 5 years post-stroke. The data to be collected will be as follows:

#### Demographics, health history, risk factors for dementia and medical treatment.

a) Sociodemographic characteristics: age, sex, marital status, handedness, education (divided into five levels), ethnic origin, native language (including bilingualism), employment status before the stroke, living environment (rural/urban). b) Family history of dementia, depression, cardiovascular and cerebrovascular diseases. c) Medical treatment, particularly medications associated with an increased risk of cognitive decline, such as those with anticholinergic effects, proton pump inhibitors, benzodiazepines, anti-seizure medications and statins, as well as all other medications recorded both prior to the stroke and at each follow-up visit throughout the study. d) Risk factors considered to be associated with an increased risk of dementia: metabolic-nutritional (hypothyroidism and hypovitaminosis B12), body mass index, cardiovascular risk factors, toxic factors (smoking, alcohol and others), traumatic brain injury, physical inactivity and level of physical activity (assessed using the Rapid Assessment of Physical Activity 1 and 2 questionnaire) [15], hearing loss, social isolation, obstructive sleep apnoea syndrome (screened using the STOP-BANG scale) [16], insomnia (assessed using the Insomnia Severity Index) [17] and vascular epilepsy after stroke.

#### Stroke characteristics and stroke recurrence.

Strokes will be classified according to type (ischemic or hemorrhagic), syndromically using the Oxfordshire Stroke Classification Project (OSCP) [18] and by etiology; in the case of ischemic strokes, the etiological Trial of ORG 10172 in Acute Stroke Treatment (TOAST) classification [19] will be used. For hemorrhagic strokes, the cause will be defined as hypertensive, suspected cerebral amyloid angiopathy according to the Boston 2.0 criteria [20] or undetermined. Stroke symptoms and severity will be assessed using the NIHSS [21] at the time of hospital arrival and upon discharge from the stroke unit. In the case of ischemic stroke, data will be collected on acute treatments (thrombolysis, thrombectomy, or both).

#### Blood samples, biobank and AD biomarkers.

Blood samples will be collected and analysed during the hospital stay and at 6 and 12 months after stroke. At baseline, all patients will be offered to donate blood samples to the Biobank of the Aragon Health System (BSSA). The following parameters will be analyzed: a) Lipid profile: total cholesterol, high-density lipoprotein, low-density lipoprotein, Apolipoprotein A1, Lipoprotein A and B, and triglycerides; b) Inflammation markers: fibrinogen, CRP, Lipoprotein-associated phospholipase A2, and myeloperoxidase; c) other risk factors: glycated hemoglobin, glucose and insulin, homeostatic model assessment insulin resistance (HOMA-IR), homocysteine, Thyroid-Stimulating Hormone, Vitamin B12, Vitamin D, folate; and, d) Genetic markers: APOE ε4 carrier status and genetic study of hereditary thrombophilias.

In patients in whom cognitive impairment is detected at 12 months, an analysis of Alzheimer’s disease biomarkers will be conducted following our local protocols.

#### Functional assessment.

The mRS scale will be used to measure the patient’s global functional status. The performance of instrumental activities of daily living will be assessed using the Spanish version of the Lawton & Brody Instrumental Activities of Daily Living Scale (IADL) [22]. The Barthel Index [23] will be used to assess the degree of independence in 10 basic ADL functions.

#### Imaging acquisition and data processing.

In all cases, a cranial CT scan will be performed within the first 24 hours after symptom onset and a cerebral MRI scan will be conducted within the first 7 days post-stroke. The MRI protocol includes an isovolumetric 3D T1-weighted sequence (1 mm resolution) and a susceptibility-weighted imaging (SWI) sequence. Specifically, on a 1.5T Siemens scanner, the following sequences are acquired: sagittal 3D T1 SPACE (1 mm isovolumetric resolution), axial proton density (PD) and T2 FLAIR (5 mm slice thickness), coronal T2 TSE (5 mm slice thickness), axial diffusion-weighted imaging (b = 1000, 5 mm slice thickness) and axial SWI (2 mm slice thickness) with 16 mm maximum intensity projection (mIP) reconstructions. In some cases, a 1.5T GE system may be used, employing equivalent sequences—though under different names—including sagittal T1 CUBE (1 mm isovolumetric resolution), axial T2 FLAIR, axial PD, coronal T2 fast spin echo (FSE) (5 mm slice thickness), and axial Susceptibility-weighted angiography (eSWAN) (2 mm slice thickness). A second brain MRI will be performed at 12 months in all patients.

A process of image harmonisation will be conducted. For each patient, the following variables will be defined according to the Neuroimaging Standards for Research into Small Vessel Disease (STRIVE-2) [24]: a) An analysis of the number and volume of lesions will be performed distinguishing between acute and chronic lesions; b) The location of the lesions and the vascular territory of acute ischemic lesions will be identified and their patterns: fragmented lesions within a vascular territory, lesions across multiple arterial territories, single cortical lesion, recent small subcortical infarcts and lacunes; c) In addition to Fazekas scale scoring, periventricular space and white matter hyperintensities will be analyzed using a recently developed pipeline initially created for the Mild Stroke Study 3 [25]. d) Cerebral microbleeds will be quantified, classifying them based on location as lobar (cortex and subcortical white matter) or deep (basal ganglia, internal and external capsules, thalamus, and posterior fossa). e) Structural images will be preprocessed using Freesurfer v7.0 (http://surfer.nmr.mgh.harvard.edu/). Freesurfer automatically processes and analyses MRI, including skull stripping, segmentation and cortical parcellation, providing regional brain volume measurements, cortical thicknesses, and intracranial volumes [26]. The hippocampal subfields will be segmented using a probabilistic ex vivo ultra-high-resolution (~0.1 mm) atlas [27]. This technique provides the volumes of 14 hippocampal subregions, including the cornu ammonis areas (CA1, CA2/3, and CA4), subiculum, presubiculum, parasubiculum, dentate gyrus (DG), hippocampus-amygdala-transition-area (HATA), fimbria, molecular layer (ML), hippocampal fissure, and hippocampal tail. The CA4, DG and ML volumes will be combined and analyzed as a single structure. Smaller subfields (< 200 mm^3^) will be removed from the analysis to avoid partial volume effects [28]. Thalamic nuclei volumes, including the Anteroventral (AV), Ventral anterior (VA), Ventral lateral anterior (VLa), Ventral lateral posterior (VLp), Ventral posterolateral (VPL), Pulvinar (Pul), Lateral geniculate (LGN), Medial geniculate (MGN), Centromedian (CM), Mediodorsal-Parafascicular (MD-Pf) in addition to the Habenula (Hb) and the Mammillothalamic tract (MTT) will be segmented using a new version of the Histogram-based polynomial synthesis (HIPS) Thalamus Optimized Multi Atlas Segmentation (THOMAS), now referred as HIPS-THOMAS [29,30]. This updated version also segments and calculates the volume of basal ganglia structures, such as claustrum, Red Nucleus, Nucleus Accumbens, Caudate, Globus Pallidus External and Internal (GPe, GPi), the whole Globus Pallidus (GPe + GPi). f) Strategic location infarcts will be defined, including the following: thalamus, frontal white matter, caudate nucleus head, anterior limb and/or genu of the internal capsule, angular gyrus, frontocingulate cortex, medial temporal area, and hippocampus.

### Neuropsychological and affective assessment

#### Screening tools.

An initial cognitive screening will be conducted at baseline and during follow-up visits using the Montreal Cognitive Assessment (MoCA ©). The Oxford Cognitive Screen (OCS) Test, in its validated Spanish version [31], will expand the domain-specific assessment during follow-up visits at 3, 6, 12 months and during subsequent annual visits. This instrument is specifically designed for cognitive impairment in stroke patients, it can be completed with one hand (taking in to account motor deficits in upper limbs), and it is inclusive for patients with aphasia and neglect.

Progressive cognitive impairment is defined as a consistent decline observed in two consecutive evaluations conducted after the third month. The distinction between MCI and dementia lies in the functional impact; dementia will be diagnosed when the cognitive deficits are sufficient to interfere with independence.

#### Affective assessment.

The Spanish version [32] of the Beck Depression Inventory (BDI-II) will be used for affective assessment. Apathy will be evaluated by means of the first question of the Frontal Behaviour Inventory [33].

#### Neuropsychological assessment battery.

At 12 months post-stroke, patients exhibiting impairment in any cognitive domain (determined through clinical assessments and screening tests) will undergo a neuropsychological battery selected by consensus among neuropsychologists with extensive experience in vascular cognitive impairment. Each section of the tests will be mapped to a specific cognitive process, based on common practice and previous work and will be standardised and adjusted to Z-scores in consideration of published normative data and fully adjusted for sex, age, and education. The scores will be categorised (normal or impaired) in accordance with different cutoff types and values.

The tests will be assigned to one of the six domains: attention and processing speed, executive function, learning and memory, language, perceptual-motor function and praxes. Based on this, domain z scores will be calculated as the standardized average of all test z scores in a particular domain. The global cognition score will be the standardised mean of all domains. From this, three types of scores will be derived: (1) a breakdown of scores by cognitive process for each individual, with dichotomisation into normal or impaired; (2) scores by cognitive domain for each individual, along with the total number of affected domains; and, (3) the overall cognitive summary score, corresponded to the average of the 6 domain scores.

The assignment of tests for the cognitive domains will be as shown in Table 1.

**Table 1:** Neuropsychological assessment battery.

Domain	Test	Purpose and Description	Reference
**Attention and Processing Speed**			
	Five-Digit Test (Reading & Counting)	Assesses sustained attention through numerical reading/ counting; Spanish version used.	Sedó, 2007
	Trail Making Test B (TMT-B)	Assesses alternating attention and cognitive flexibility.	Partington, 1949
	Trail Making Test A (TMT-A)	Measures processing speed and visual scanning; adapted Spanish norms applied.	Partington, 1949
	Symbol Digit Coding	Tests visual attention and processing speed; Spanish normative data used.	Smith, 1982/ Peñalver et al., 2018
	Five-Digit Test (Choosing)	Evaluates selective attention via numerical discrimination tasks.	Sedó, 2007
**Executive Function**			
	Digit Span test (WAIS-III/IV)	Verbal working memory via forward and backward repetition; Spanish version.	Wechsler, 1955
	BADS (Key Search)	Assesses planning, reasoning and problem-solving in ecological scenarios.	Wilson et al., 1996
	Wisconsin Card Sorting Test -WCST- (number of Categories, % of Non-Perseverative Errors)		Heaton, 1981
	WCST (Perseverative Errors and Response)	Cognitive flexibility, abstraction, and error correction; adapted norms applied.	Heaton, 1981
	Phonemic Verbal Fluency (PMR)	Measures verbal fluency initiation using P, M, R (Spanish adaptation of FAS).	Peña-Casanova et al., 2009
	Five-Digit Test (Inhibition)	Evaluates inhibitory control in interference conditions.	Sedó, 2007
**Learning and memory**			
	TAVEC – Spain-Complutense Verbal Learning Test	Spanish verbal list-learning task for recall (immediate/delayed), recognition, and discrimination.	Benedet & Alejandre, 1998
**Language**			
	Boston Naming Test	Visual object naming test adapted to Spanish language/culture.	Kaplan et al., 1983/Cuetos et al., 1996
	Barcelona Test – Written Commands	Assesses comprehension of written verbal instructions;	Peña-Casanova et al., 2009
**Perceptual-Motor**			
	Barcelona Test – Overlapping Figures	Screens for perceptual-motor discrimination; Spanish adaptation used.	Peña-Casanova et al., 2009
**Praxes**			
	Unilateral/Bilateral Praxes	Evaluates ideomotor praxis with both unilateral and bilateral commands.	Peña-Casanova et al., 2009
**Neuropsychiatric**	Neuropsychiatric Inventory Questionnaire	Informant-based screening of neuropsychiatric symptoms; Spanish validated version used.	Kaufer et al., 2000/Boada et al., 2002

### Study plan

The study timeline is presented in Table 2

**Table 2:** Assessment timeline.

Assesment	T0	T1	T2	T3	T4-7
SS-IQCODE	✓				
Demographics	✓				
Health history	✓	✓	✓	✓	✓
Risk factors for cognitive impairment	✓	✓	✓	✓	✓
STOP-BANG questionnaire		✓	✓	✓	✓
Insomnia Severity Index- ISI		✓	✓	✓	✓
Rapid Assessment of Physical Activity – RAPA 1 + 2		✓	✓	✓	✓
Family history	✓				
Medication	✓	✓	✓	✓	✓
Blood Samples	✓		✓	✓	
Biobanking	✓				
Stroke characteristics	✓				
National Institute of Health Stroke Scale – NIHSS	✓				
Magnetic Resonance Imaging	✓			✓	
Stroke recurrence	✓	✓	✓	✓	✓
Modified Rankin Scale- mRS	✓	✓	✓	✓	✓
Barthel Index	✓	✓	✓	✓	✓
Lawton instrumental activities of daily living scale –IADL	✓	✓	✓	✓	✓
Montreal cognitive assessment- MOCA	✓	✓	✓	✓	✓
Oxford Cognitive Screen OCS		✓	✓	✓	✓
Beck Depression Inventory II – BDI-II		✓	✓	✓	✓
Frontal Behaviour Inventory – FBI (apathy)		✓	✓	✓	✓
Neuropsychological Assessment^*^				✓	✓
The Neuropsychiatric Inventory Questionnaire [34]				✓	✓
AD Biomarkers^**^				✓	✓

T0: Baseline, T1: Three months follow-up, T2: 6 months follow-up, T3: 12 months follow-up T4: Annual Follow-Up.

* With patients who show impairment in screening tests

** With patients in whom impairment is confirmed through the neuropsychological assessment.

### Data analysis

A descriptive analysis of the sample will be performed during the baseline period corresponding to the initial phase of the study. This will involve summarising the characteristics of the sample population to provide a clear understanding of its composition before further observations. Qualitative variables at the baseline of the study will be described through frequency distributions, expressed as percentages for each category. Normality of the variables will be assessed using the Shapiro-Wilk test (or similar) and Q-Q plots. Variables will be described using central tendency measures (mean or median), non-central position measures (first and third quartiles), and dispersion measures (standard deviation and/or interquartile range) in terms of the data distribution.

To determine the early and late incidence of PSCI, the outcome variable will be the diagnosis of PSCI, coded as a dichotomous variable (yes/no) at each evaluation time point (3, 6, and 12 months). The cumulative incidence of PSCI will be calculated at each of these time points, defined as the number of new cases identified among patients under follow-up who did not present it at the previous evaluation, divided by the total number of evaluable patients at that time.

To identify predictors of cognitive impairment and factors associated with its progression, univariable and multivariable binary logistic regression models will be used to determine independent predictors of PSCI at each evaluation point (3, 6, and 12 months). In the univariable analysis, explanatory variables will include demographic and clinical characteristics, comorbidities, pharmacological variables, risk factors, and biomarkers. For the multivariable analysis, potential interactions between variables will be explored, and model fit and collinearity will be assessed. Results will be presented with corresponding odds ratios (ORs), 95% confidence intervals (CIs), and p-values to quantify the strength and significance of associations.

All statistical tests will be two-tailed, with a significance level set at p < 0.05. Effect sizes will be reported for each comparison. When multiple comparisons are performed, p-values will be adjusted using appropriate correction methods.

To explore the existence of distinct patient profiles more prone to developing PSCI, unsupervised learning techniques will be applied, including clustering methods (k-means and hierarchical clustering) and principal component analysis (PCA), to identify subgroups of patients with similar clinical, neuropsychological, or biological patterns. The resulting groups will be clinically characterized and compared in terms of PSCI prevalence and progression using a combination of descriptive, comparative, and inferential methods, depending on the nature of the variables. Quantitative variables will be analyzed using means and standard deviations per group. Differences between groups will be assessed using ANOVA (if normality is met) or the Kruskal-Wallis test (if not). If global tests are significant, post-hoc comparisons (Tukey or Dunn) will be performed. For categorical variables, frequencies and percentages will be calculated by group, and the Chi-square or Fisher’s exact test will be used to compare proportions. To compare groups with respect to PSCI and its progression, PSCI will be treated as a dichotomous outcome (yes/no at different time points), and rates will be compared using the Chi-square test for global differences, followed by pairwise comparisons with correction for multiple testing (Bonferroni or Holm).

To assess PSCI progression, an ordinal variable will be created (no PSCI → mild → moderate → dementia), and appropriate ordinal regression models will be applied if assumptions are met. Linear trend will be tested using the Cochran-Armitage trend test, and Kruskal-Wallis tests will be used to compare cognitive score medians across groups. Finally, multivariable models (e.g., multinomial logistic regression) will be used to estimate the probability of group membership based on clinical features and PSCI status, and discriminant analysis will be performed to evaluate whether the identified profiles can accurately predict PSCI presence and progression.

To define the neuropsychological profile of patients with PSCI, assess the relationship between these profiles and stroke characteristics, and explore whether specific patterns predict progression and coexistence with AD, a more exploratory and descriptive approach will be applied. Key variables of interest will include performance on neuropsychological tests across cognitive domains and processes, stroke-related characteristics, and the presence of biomarkers compatible with AD. Analytical strategies will build upon the descriptive and hypothesis-testing techniques outlined in previous objectives. In addition, dimensionality reduction methods such as exploratory factor analysis (EFA) or principal component analysis (PCA) will be used to identify common cognitive patterns (e.g., amnestic, frontal, mixed profiles). Associative techniques will also be employed, including Pearson or Spearman correlations between cognitive performance and stroke volume/location. Furthermore, multiple linear regression models will be used to predict cognitive scores based on clinical and neuroimaging variables.

To determine the prevalence of depression and apathy after stroke and to identify associated predictive factors, analytical methods similar to those described in previous objectives will be applied to new outcome variables. The dependent variables will be the presence of depression (both as a dichotomous and categorical variable) and/or apathy (dichotomous), while independent variables will include age, sex, PSCI status, functional status, among others. Prevalence will be estimated at each follow-up time point using point estimates and 95% CI. For comparative analysis, hypothesis testing will be conducted to compare patients with and without affective symptoms—following a similar approach as used for PSCI analysis. Separate multivariable regression models will be constructed for depression and apathy, using binary logistic regression to explore independent predictors.

To assess whether predictive factors differ between early and late cognitive impairment, a more structured multinomial and comparative approach will be implemented, expanding upon the previously described techniques. A categorical dependent variable with three levels will be created: no PSCI, early PSCI (3–6 months), and late PSCI (≥12 months). A multinomial logistic regression model will be applied to identify differential predictors across these groups, including the coexistence of AD and/or vascular pathology. Both global model significance and pairwise category comparisons will be evaluated. Group differences will be assessed using ANOVA or the Kruskal-Wallis test for continuous variables and the Chi-square test for categorical variables. Multinomial logistic regression models will estimate the ORs for belonging to the early or late PSCI groups versus no PSCI, as a function of various predictors (e.g., age, stroke characteristics, ApoE4 status…). Additionally, interaction analyses will be conducted to explore whether certain predictors exert a stronger influence on late PSCI compared to early PSCI.

To develop and validate a predictive model for PSCI, techniques similar to those described in Objective 3 will be used, but with a focus on individual-level prediction (risk modeling) rather than association alone. Validation strategies and machine learning approaches will be incorporated. The dependent variable will be the diagnosis of PSCI (binary: yes/no), and a broad set of clinical, neuropsychological, and biological predictors will be included as independent variables. A predictive model will be developed using logistic regression, with the dataset split into a training set (70–80% of the sample) and a validation set (20–30%). K-fold cross-validation will be applied to reduce overfitting. In addition to logistic regression, more complex models such as Random Forest, Gradient Boosting, and classification trees will be explored. Model performance will be evaluated using metrics such as accuracy, sensitivity, specificity, and the area under the ROC curve (AUC-ROC). The discriminative capacity of each model will be compared, with preference given to those that are both parsimonious and clinically interpretable.

### Ethical aspects

This project, in its version v2 (October 2021), was reviewed and approved by the Research Ethics Committee of the Community of Aragón (CEICA) in its meeting on November 3rd, 2021 (Act No. 20/2021). CEICA receives an annual progress report detailing the study’s development. Additionally, each participating institution has obtained approval from its respective local Ethics Committee. Written informed consent was obtained from all participants prior to inclusion in the study.

## Discussion

This paper outlines the protocol of the ICTUSCOG study, an ongoing prospective cohort study that will follow participants for five years. It will be the first study to quantify and report cognitive impairment in a large multicenter Spanish cohort following nondisabling stroke.

Our primary objective is to integrate cognitive and affective assessments into routine stroke care, from the acute phase through long-term follow-up. ICTUSCOG will support the development of a structured care pathway and provide insight into PSCI and its impact on quality of life, particularly in middle-aged adults.

The main focus will be on identifying predictors of impairment, determinants of progression, and risk profiles through the combination of biomarkers and clinical markers. This approach will allow exploration of the relationship between cognition and factors such as APOE status, inflammatory response, chronic inflammation, insomnia, obstructive sleep apnoea, physical activity, social interaction, hearing loss and stroke recurrence, among others. Brain MRI analyses will be integrated with advanced techniques to identify neuroimaging biomarkers. This will clarify the role of stroke as a trigger for neurodegeneration and the contribution of comorbidities and pre-existing pathology to cognitive decline.

The RICORS ICTUS network has facilitated implementation across diverse centers, enriching our cohort with unique factors such as bilingualism.

The neuropsychological battery, designed by experts in VCI, emphasizes the importance of individualised analysis of cognitive processes to detect subtle impairments in specific cognitive domains that often go unnoticed in global scores. The presence of affective symptoms following stroke, even in individuals without prior history, is recognised as part of an adaptive process but also as a feature intrinsic to stroke pathology that may amplify functional limitations and reduce quality of life, especially in working-age adults.

Controlling vascular risk factors is essential to limit further decline. Achieving secondary prevention targets will help prevent recurrent strokes and manage small vessel disease, key contributors to long-term outcomes.

PSCI assessment is usually at least 3–6 months after stroke, allowing for resolution of transient complications. [35] However, late-onset PSCI remains a concern, underscoring the need for extended follow-up. Prior studies often lacked this, failed to exclude patients with pre-stroke dementia or have not focused on individuals who recover without apparent physical sequelae or with only mild deficits. We acknowledge that, given the high prevalence of patients with these characteristics, long-term follow-up for all of them may not be feasible. Therefore, ICTUSCOG aims to advance the understanding of PSCI trajectories by developing a predictive tool that integrates risk factors and markers. This tool will support early identification of individuals at risk during the acute stroke phase, enabling timely interventions and strategies to mitigate the burden of PSCI.

### Limitations

The ICTUSCOG study has certain limitations. The neuropsychological assessment is planned to be administered within 60 minutes, however, in many cases, the evaluation may take 75–90 minutes and its application is limited to Spanish-speaking populations. Furthermore, the battery is not fully standardised as a single unit and this may restrict access to the tests. Combined with the limited availability of neuropsychologists in routine clinical practice at other centres, this could limit the generalizability of the findings. Additionally, the routine use of 1.5-tesla MRI in our hospital and the timing for the follow-up neuroimaging at 12 months may not be strictly adhered to due to waiting list constraints. Some limitations could arise from not considering relevant variables that are difficult to control, for example, socioeconomic status, diet, and treatment adherence.

### Dissemination plan

Dissemination of the study results will aim to ensure maximum visibility. The findings will be published in open-access scientific journals and presented at national and international conferences and seminars. Our goal with this project is to propose and implement a care protocol for these patients. We also aim to disseminate the results to raise awareness about the cognitive-behavioural sequelae of stroke, emphasising the importance of cognitive decline prevention at all stages of life, as advocated by the WHO, European Academy of Neurology and AAN.

### How potential changes in the study will be approached

Any modification to the protocol such as modifications to the selection criteria or changes to the assessment tool will be reported immediately to the bioethics committee for approval.

## Conclusions

ICTUSCOG will provide a renewed perspective, building on current knowledge through the application of advances in biomarkers and neuroimaging analysis. It will offer valuable insights into the pathophysiological basis of cognitive impairment in patients who suffer a stroke that is initially deemed non-disabling and will help develop tools for identifying patients at risk. In a resource-limited healthcare system, the implementation of these tools creates an efficient therapeutic window for early intervention which could potentially mitigate progression and associated complications. In the future, this work will serve as a foundation for developing prevention strategies and enhancing the rehabilitation process for these patients. The results will contribute to the establishment of care pathways and the potential development of future interventional studies targeting PSCI.
